# Simultaneous debridement, Ilizarov reconstruction and free muscle flaps in the management of complex tibial infection

**DOI:** 10.5194/jbji-6-63-2020

**Published:** 2020-12-22

**Authors:** Max Mifsud, Jamie Y. Ferguson, David A. Stubbs, Alex J. Ramsden, Martin A. McNally

**Affiliations:** 1 The Oxford Bone Infection Unit, Nuffield Orthopaedic Centre, Oxford, University Hospitals NHS Foundation Trust, Windmill Road, Oxford, OX3 7HE, UK

## Abstract

Chronic bone infections often present with complex bone and soft tissue loss. Management is difficult and commonly delivered in multiple stages over many months. This study investigated the feasibility and clinical outcomes of reconstruction in one stage.

Fifty-seven consecutive patients with chronic osteomyelitis (n=27) or infected non-union (n=30) were treated with simultaneous debridement, Ilizarov method and free muscle flap transfer. 41 patients (71.9 %) had systemic co-morbidities (Cierny-Mader group Bs hosts). Infection was confirmed with strict criteria. 48 patients (84.2 %) had segmental defects.

The primary outcome was eradication of infection at final follow-up. Secondary outcomes included bone union, flap survival and complications or re-operation related to the reconstruction.

Infection was eradicated in 55/57 cases (96.5 %) at a mean follow-up of 36 months (range 12–146). No flap failures occurred during distraction but 6 required early anastomotic revision and 3 were not salvageable (flap failure rate 5.3 %).

Bony union was achieved in 52/57 (91.2 %) with the initial surgery alone. After treatment of the five un-united docking sites, all cases achieved bony union at final follow-up.

Simultaneous reconstruction with Ilizarov method and free tissue transfer is safe but requires careful planning and logistic considerations. The outcomes from this study are equivalent or better than those reported after staged surgery.

## 1 Introduction

Chronic bone infection results in bone destruction and often disrupts the soft tissues. This is common after open fractures, particularly in the lower tibia, where soft tissue reconstruction options are limited (Olesen et al., 2015; Metsemakers et al., 2017). Successful treatment relies on adequate debridement, culture specific antibiotics, dead space management, bony stabilisation and definitive soft-tissue coverage (Cierny and DiPasquale, 2006; McNally et al., 2016; Conterno and Turchi, 2013). A specialist multi-disciplinary approach to diagnosis and treatment is also advocated (Cierny and DiPasquale, 2006; McNally et al., 2016; Metsemakers et al., 2019; Ziran et al., 2003; Bose et al., 2015; Chan et al., 2019).

Many centres perform free flap coverage as part of staged surgery, particularly when distraction osteogenesis is planned. This has been recommended in open tibial fractures, with a short interval between the stages (British Orthopaedic Association and British Association of Plastic, Reconstructive and Aesthetic Surgery, 2020; National Institute for Health and Care Excellence, 2020; Gopal et al., 2000). However, the most challenging cases include those with segmental bone involvement and major soft tissue loss, together with established infection. These cases have traditionally been treated with a staged approach, addressing the infection first, usually with serial debridements and antimicrobial therapy. The soft tissues may be closed initially or later, using a free flap, with or without a period of open wound management. Finally, the bone is addressed with Ilizarov bone transport, cancellous bone grafting or by incorporating vascularised bone in the tissue transfer (McNally et al., 1993; Lowenberg et al., 1996; Metsemakers et al., 2019; Masquelet et al., 2000; Sanders and Mauffrey, 2013). This staged concept has been reported with good results, but requires a prolonged period in treatment, with multiple operations and significant co-morbidity for patients.

Improved microsurgical techniques have demonstrated successful outcomes when using free tissue transfer, with increased rates of infection-free union (Chan et al., 2019; Lowenberg et al., 1996; Mathews et al., 2015). However, microvascular reconstruction in this group is perceived as difficult, due to perivascular fibrosis, vascular spasm, the potential for previous vessel injury following trauma, as well as the challenges of access when using an external fixator (Chan et al., 2019; Li et al., 2020).

Undertaking bone excision, Ilizarov frame stabilisation and free tissue transfer as a single procedure may be logistically and technically demanding but few studies have considered this (Lowenberg et al., 1996; Chim et al., 2011; McKee et al., 2008; McNally et al., 2017). Lowenberg et al. (1996) advocated leaving the definitive reconstruction until after serial debridements, to eradicate infection and only 13 of 36 cases had the flap and frame applied in the same operation. McKee et al. (2008) reported on 8 infected tibial non-unions with 5 free flaps transferred at the same time as application of an Ilizarov frame. Chim et al. (2011) treated 18 infected tibial non-unions with a staged approach but had a 10 % flap failure rate and the patients required multiple operations.

This paper presents a longitudinal observational cohort study of a consecutive series of patients with segmental infection of the tibia and major soft tissue compromise, treated with debridement, Ilizarov reconstruction and free flaps in a single-stage procedure. The practical implications and outcomes for the patients are reported.

## 2 Methods

### 2.1 Inclusion criteria

Patients were included if they had surgical management of chronic osteomyelitis or infected non-union of the tibia with simultaneous application of an Ilizarov ring fixator and a free muscle flap. All patients had major soft tissue defects which could not be closed directly, by local tissue transposition or by limb shortening.

All patients were assessed in a multidisciplinary clinic, comprising orthopaedic and plastic surgeons, infectious disease physicians and a specialist Ilizarov nurse practitioner. The diagnosis of osteomyelitis or infected non-union was confirmed with clinical, microbiological and histology criteria as previously described (BMJ, 2020; Govaert et al., 2020; Morgenstern et al., 2018).

Patients were excluded if they had infection outside the tibia, did not meet the diagnostic criteria for osteomyelitis or infected non-union, or were under 18 years old.

### 2.2 Data collection

Prospective data was collected on patient comorbidities, associated limb deformity, intraoperative microbiological and histological sampling, Ilizarov strategy used and complication rates and outcomes during the treatment period and follow-up.

Cases were classified using the Cierny-Mader classification (Cierny et al., 2003) for osteomyelitis and Weber and Cech (1976) for infected non-unions. Ilizarov techniques were categorized on how the frame was used into: (1) protective, if used purely to protect the bone from fracture, or stabilize a simple fracture without major bone loss following debridement, (2) monofocal compression for mobile non-unions, (3) monofocal distraction for stiff non-unions, (4) bifocal acute shortening and relengthening (ASR) and (5) bone transport (McNally et al., 2017; Sigmund et al., 2020; Tetsworth et al., 2017; Ferreira et al., 2015).

### 2.3 Surgical management

All patients had excision of the non-viable infected bone using a standard technique previously described (McNally et al., 2016; Ferguson et al., 2014). Multiple intra-operative microbiological and histological samples were collected before antibiotics were given, using a validated protocol to improve diagnostic accuracy (Govaert et al., 2020; Morgenstern et al., 2018; Hellebrekers et al., 2019). Sinus tracts and implants were removed. Intramedullary nails were removed and the canal reamed. Excision was complete when only healthy bleeding bone was present. A thorough wound irrigation was performed with 0.05 % aqueous chlorhexidine to remove debris and residual planktonic bacteria. If required, a local antibiotic carrier was used to fill any bone voids. In medullary canals, Herafill with Gentamicin pellets were inserted (Heraeus Medical GmbH, Wehrheim, Germany). Cerament with Gentamicin or Vancomycin (Bonesupport AB, Lund, Sweden) was used to fill cortico-medullary defects, such as empty screw holes, or cortical bone windows. When a segmental excision was performed, the bone gap was left free of local antibiotics, to prevent residual material blocking bone transport and docking. Non-union mobility was assessed following debridement and judged to be “stiff” if there was angular bending of less than 7${}^{\circ }$ and axial motion of <5 mm on loading (Ferreira et al., 2015).

**Figure 1:**
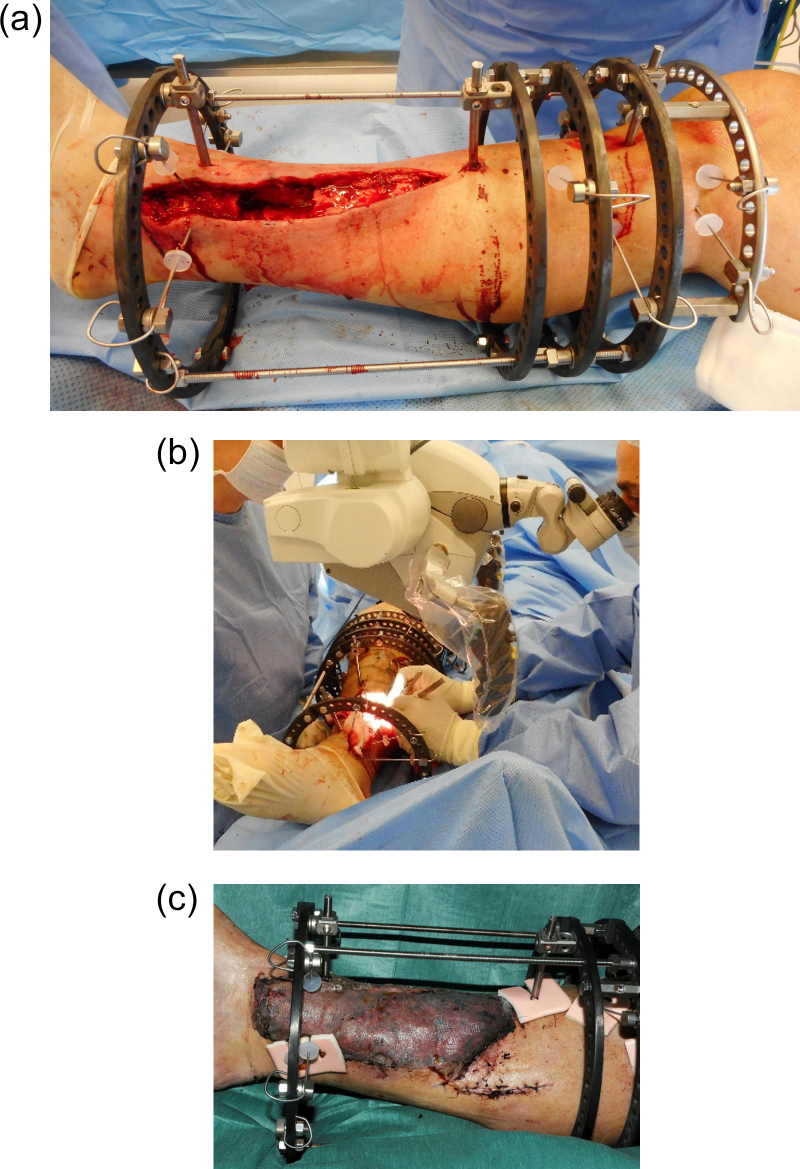
This patient had a closed lower tibial fracture, treated with a plate. He presented at 5 months after injury with wound breakdown and an infected non-union. The infected segment has been excised **(a)** and a modified Ilizarov frame applied to stabilize the tibia and allow bone transport. The modified frame **(b)** facilitates unobstructed access to the posterior tibial vessels for microvascular anastomosis. **(c)** Latissimus dorsi free flap in situ, 7 d after surgery. The distal fixator pins have been placed around the edges of the flap, avoiding the pedicle. Distal progression of the proximal bone transport rings will not impinge on the postero-medial anastomosis.

Simultaneous use of ring fixators and vascularised muscle flaps required careful preoperative planning between the orthopaedic and plastic surgical teams. Plastic surgeons need to be comfortable during microsurgery, without parts of the frame obscuring vision or limiting hand movements. Pre-operative angiograms were performed when distal pulses were absent. The site of the vascular anastomosis was decided and a frame designed to allow sufficient access for the microsurgery (Fig. 1a, b). Wires were placed away from the intended pedicle of the flap to ensure that during bone transport or lengthening, they will not kink or cross the pedicle. Wires were usually sited around the edge of the soft tissue defect, but can be passed through muscle flaps after transfer, providing the pedicle is avoided (Fig. 1c).

**Figure 2:**
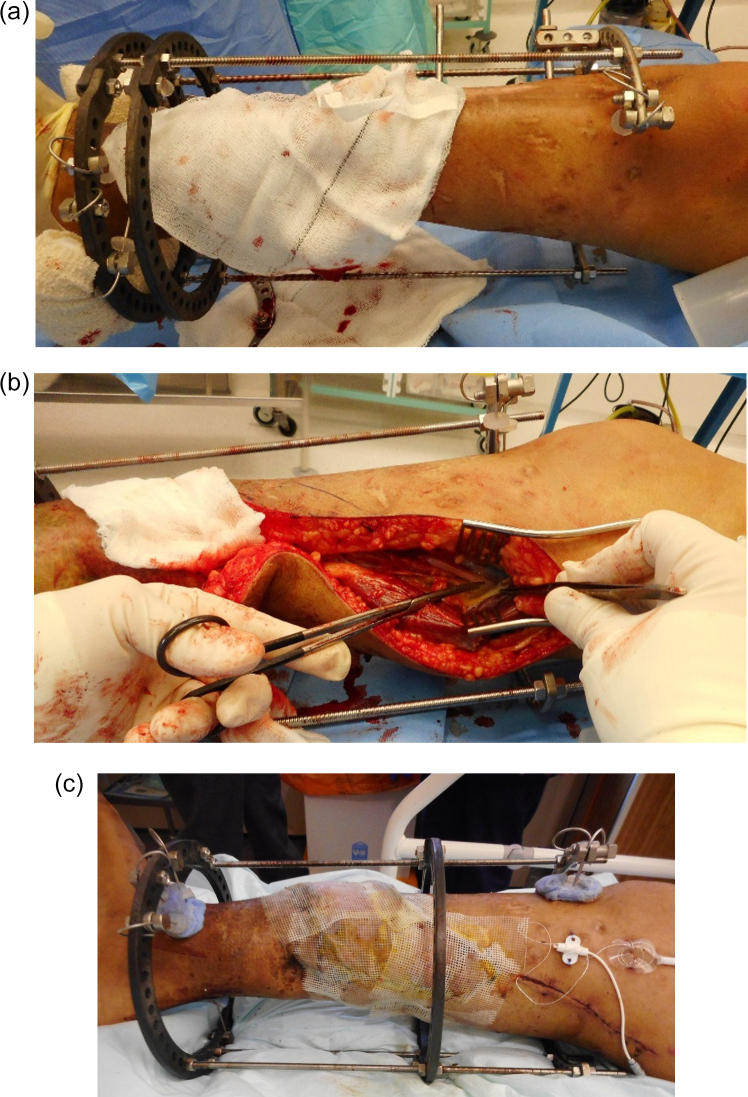
This young man suffered a fracture through an area of chronic osteomyelitis, after a previous Ilizarov bone transport. It was treated conservatively but developed a hypertrophic non-union with shortening. He was referred to us with a painful, stiff non-union and a large anteromedial ulcer. A simplified Ilizarov frame with a partial proximal ring has been applied to temporarily stabilize the tibia **(a)**, after excision of the ulcer and dead bone at the fracture site. The central ring of the frame has been moved distally (by sliding along the rods) and the medial rods are absent. The only patent recipient vessels were in the proximal part of the calf. This highly modified frame allowed good access for microsurgery **(b)**. After surgery **(c)**, the central ring has been moved to its proper position on the diaphyseal half pin. Further rods and hinges are applied later to improve stability. Temporarily leaving out the final rods, and ring segments allows continued access to the anastomosis, if early problems arise. These must be applied before mobilisation and before monofocal distraction begins.

It was often necessary to leave out rods or to move rings temporarily to improve access. These were added later, before mobilization or transport. Partial rings can be helpful to allow access but must be used with caution to prevent loss of construct stability (Fig. 2a–c).

The free flap anastomosis was carried out using a microscope and occasionally long micro instruments were needed. In more recent cases a Flow Coupler device (Synovis Life Technologies, St Pauls, Minnesota, USA) was used for the venous anastomosis. The muscle flap was covered with a hand fenestrated split skin graft.

### 2.4 Antibiotic management

Antibiotics were stopped two weeks before surgery. Following intraoperative sampling, all patients were given empirical intravenous Vancomycin and Meropenem. Culture-specific systemic antibiotics continued for between 6 and 12 weeks.

### 2.5 Outcome measures

The primary outcome was the rate of infection recurrence at final follow-up. Failure of treatment was defined as having positive cultures from aspiration or biopsy; recurrent sinus formation; further surgery performed for infection; or any patient requiring antibiotic treatment for persisting symptoms (McNally et al., 2016; Ferguson et al., 2014).

Secondary outcome measures included bone union, flap survival and re-operation due to complications related to the reconstruction and death.

Final outcome, in all patients, was assessed by two independent assessors (Max Mifsud and Jamie Y. Ferguson), who contacted the patients and reviewed the final clinic attendance records.

**Table 1:** Classification of patients by co-morbidities and Non-union type.

Patient Classification by Cierny & Mader Group and Bone Viability					
	${}^{a}$C-M 3 B${}^{l}$	${}^{b}$C-M 3 B${}^{\mathrm{ls}}$	C-M 4 B${}^{l}$	C-M 4 B${}^{\mathrm{ls}}$	Total
Initially Intact Bone	2	6	5	14	27
Non-union	0	0	9	21	30
Total	2	6	14	35	57
Non-union Type by Weber & Cech Class${}^{c}$					
B	0	0	1	2	3
C	0	0	0	2	2
D	0	0	2	3	5
E	0	0	4	7	11
F	0	0	2	7	9
Total	2	6	14	35	57

${}^{a}$ All patients are at least Cierny-Mader Group B${}^{l}$ due to the local compromise of the soft tissues. All non-unions must be at least C-M Type 4 (diffuse or segmental involvement). ${}^{b}$ Group B${}^{\mathrm{ls}}$ implies the presence of local and systemic compromise. Systemic co-morbidities included tobacco smoking, diabetes mellitus, malignancy, peripheral vascular disease, ischaemic heart disease and sickle cell anaemia. ${}^{c}$ There were no Class A patients in this series. Class B and C non-unions are viable; class D, E and F are non-viable.

## 3 Results

Fifty-seven consecutive patients (45 men) (mean age 49.3 years; range 18.9–84.6) were treated. Forty-one (71.9 %) of the cases had at least one significant systemic co-morbidity (Cierny-Mader Group Bls, compromised hosts) (Cierny et al., 2003). Mean follow-up was 35.1 months (range 12 months–12.2 years). Table 1 summarizes the classification of patients.

### 3.1 Ilizarov method and muscle flaps

Thirty cases presented with an infected non-union and a further 18 cases, with initially intact bones, required segmental resection to eradicate infection. Bony reconstruction was achieved with monofocal compression in 15 cases, monofocal distraction in 15, bifocal ASR in 9, bone transport in 9, and a protective frame in 9 cases. Eleven patients, with severe distal tibial infection, had an ankle fusion included in the reconstruction. Nine cases with an associated angular deformity were corrected during the same frame treatment. Thirty-five patients had local antibiotics implanted to fill medullary or cortical bone defects.

The muscle flaps transferred were gracilis (n=48, including one in combination with a gastrocnemius flap), latissimus dorsi (n=8), and rectus abdominis (n=1).

**Table 2:** Microbiological culture results in the series.

Organism			Number of cases
Polymicrobial			23
Culture negative			6
Monomicrobial			27
	*Staphylococcus aureus*		9
		MRSA	2
		CoNS	4
	*Pseudomonas* spp.		5
	Diphtheroids		2
	*E. coli*		1
	*Enterobacter cloacae*		2
	*Serratia marcescens*		2

MRSA Methicillin-resistant *Staph. aureus*CoNS Coagulase-negative Staphylococci

**Table 3:** Culture results of polymicrobial infections (n=23).

Organism	Number of cases
*Staphylococcus aureus*	12
CoNS	6
MRSA	1
*Streptococcus* spp.	9
*Enterococcus* spp. (Vancomycin Resistant Enterococcus)	7 (1)
*Pseudomonas* spp.	6
*Enterobacter cloacae*	3
*E. coli*	3
Proteus	2
Citrobacter	2
Coliforms	1
Anaerobes	2
*Pasteurella* spp.	1

### 3.2 Microbiology

Results from deep tissue sampling are shown in Tables 2 and 3. Twenty-three cases (40.4 %) cultured more than one organism. The most common pathogen was staphylococcus (n=34) including coagulase negative staphylococci (n=10), methicillin resistant *Staphylococcus aureus* (MRSA) (n=3), and methicillin sensitive *Staphylococcus aureus* (MSSA) (n=7). Six patients were culture negative (infection confirmed on histology or with a draining sinus).

### 3.3 Frame duration

Mean frame time was 6.1 months (range 1.9–13.6). Protective frames had the shortest frame time (3.9 months, range 1.9–6.5) followed by monofocal compression (5.0 months, 2.9–9.7), monofocal distraction (5.9 months, 2.8–12.3), ASR (7.5 months, 3.9–13.6) and bone transport (9.4 months, 6.5–13.1). Mean re-lengthening in the ASR frames was 3.6 cm (range 2–6.5); mean frame index 2.1 months per cm (range 1.5–2.9). Bone transport cases had a mean defect size of 6.1 cm (range 4.5–10 cm); mean frame index 1.5 months per cm (range 0.9–2.1).

### 3.4 Recurrence of infection

Two patients suffered recurrent infection (3.5 %). One developed a discharging sinus overlying a pin site distant from the previous non-union, 14.1 months after index surgery. This spontaneously resolved without treatment. The second case presented after 74 months with an ulcer affecting the proximal margin of the old gracilis flap at a previous pin site, again distant from the previous osteomyelitis site. At surgery he had dead cortical bone with no intramedullary extension. The ulcer was treated with a new gracilis free muscle flap. Culture isolated a Pseudomonas species which was different from the microbiology found at index procedure. Both remained infection-free during further follow-up.

### 3.5 Bone Union

Union was achieved in 52/57 cases (91.2 %) with index surgery. In 2/5 of the un-united cases, a docking site non-union was treated with an intramedullary nail. Another case presented with varus collapse of the regenerate, successfully treated with a second Ilizarov distraction and 3.5 cm lengthening to restore mechanical axis. Two further cases required internal fixation of the regenerate with plating. All 5 cases went on to infection-free union, at final follow-up.

### 3.6 Complications

#### 3.6.1 Flap complications

**Figure 3:**
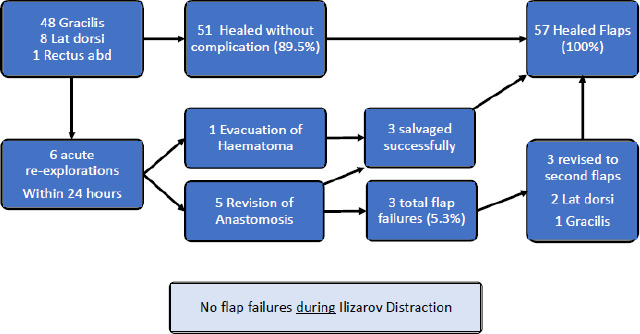
Outcome of the Free Muscle Flaps in all 57 patients.

Six cases required urgent flap re-exploration; five for revision of the anastomosis and one for evacuation and irrigation of haematoma. Three flaps were successfully salvaged, giving a flap failure rate of 5.3 %. Figure 3 presents the outcome of all flaps.

**Figure 4:**
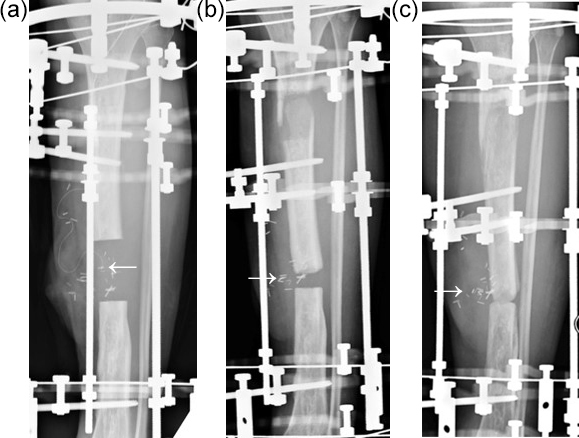
This open tibial fracture was initially treated with an intramedullary nail but developed a large segment of infected dead bone. He had a segmental excision (6 cm) with a free gracilis muscle flap **(a)**. The white arrow indicates the position of the vascular anastomosis on the underside of the flap (identified by the metallic flow coupler). During bone transport, the muscle flap is gradually pushed out of the bone defect by the advancing transport segment and the vascular pedicle is displaced distally and medially **(b)**. At docking, the anastomosis has moved medially **(c)**. The vascular pedicle has lengthened by about 5 cm during transport. The flap remained well perfused and healthy.

There were no other partial or total flap failures in any patient. No flaps developed compromise during relengthening, angular correction or bone transport. Vascular pedicles lengthened or moved during distraction without difficulty (Fig. 4).

#### 3.6.2 Fracture

Four patients (7.0 %) sustained fractures of the affected limb between one month and seven years after surgery. Three were treated successfully with walking casts. The last case sustained a fracture through the docking site one month after frame removal due to a fall downstairs. This was treated with an intramedullary nail and achieved uneventful union. No patient with a fracture had recurrence of infection.

#### 3.6.3 Amputation

One patient developed a squamous cell carcinoma at the excision site 13 months following successful healing. This presented as a new ulcer that was biopsied. She underwent a curative above knee amputation.

### 3.7 Other additional surgical intervention

#### 3.7.1 Planned surgery during initial Ilizarov frame time

Nine cases had a planned delayed corticotomy. In 6/9 this was for deformity correction and was delayed due to the Centre of Rotation of Angulation (CORA) (Paley, 2002) being close to the osteomyelitis excision site. In 3/9 cases, the corticotomy was for bone lengthening. Corticotomy was delayed due to the extent of infection from an intramedullary nail in one case, due to the close proximity of the proposed corticotomy site to the flap pedicle in one case, and due to one patient's wish to see if they could tolerate the leg length discrepancy expected following surgery. Nevertheless, of the 19 cases treated with ASR or bone transport, 16 (84.2 %) had their corticotomy performed at index procedure with no complication arising.

#### 3.7.2 Unplanned Surgery

Eight patients had unplanned operations during frame treatment. Only five cases had docking site surgery (refreshing with bone grafting or bone morphogenetic protein) to facilitate union. Three frame adjustments or realignments were undertaken. One patient required a repeat fibular osteotomy for premature fusion.

#### 3.7.3 Outcome following index procedure

The primary outcome measure of eradication of infection was achieved in 55/57 (96.5 %) of cases. Considering all unplanned interventions and all complications during follow-up, only 36 cases (63.2 %) achieved infection-free bony union following index procedure alone without any event. However, at final follow-up, all cases, aside from the single case requiring amputation due to carcinoma, had achieved bony union with no sign of infection (56/57 (98.2 %) (Table 4).

**Table 4:** Breakdown of outcomes for the different types of Ilizarov method used.

Ilizarov Method	Cases	Flap	Planned	Unplanned	Unplanned	Refracture	Recurrence of	Final Infection
		failure	operation during	operation during	operation after	after frame	infection	free union
			Ilizarov frame	Ilizarov frame	Ilizarov removal	removal		(%)
Protective	9	1	0	0	0	1	0	88.9${}^{d}$
Monofocal compression	15	1	0	0	1	0	1	100
Monofocal distraction	14	0	6	1	1	1	1	100
Bifocal, acute shortening & relengthening	10	0	2	3	0	1	0	100
Bone Transport	9	1	1	5	3	1	0	100
Total (%)	57	3 (5.3)	9${}^{a}$ (15.8)	9${}^{b}$ (15.8)	5${}^{c}$ (8.8)	4 (7.0)	2 (3.5)	57${}^{d}$ (98.2)

${}^{a}$ all nine planned procedures were delayed corticotomies; ${}^{b}$ including docking site procedures, realignments and fibular osteotomy; ${}^{c}$ all procedures to secure bony union (2 IM nails, I Ilizarov, 2 plates); ${}^{d}$ the single patient who required amputation due to squamous carcinoma is not included as a final infection-free union.

During follow-up three patients died of unrelated causes at a mean of 3.8 years following index surgery (range 3.1–4.2 years).

## 4 Discussion

This series reports good outcomes for infection treatment at the more severe end of the spectrum of complexity, with almost three-quarters exhibiting significant medical co-morbidities, 84.2 % with an infected segmental defect, and 19.0 % presenting with adjacent septic arthritis. All had major soft-tissue compromise, requiring free tissue transfer. Most importantly there were no late flap failures related to frame adjustments or bone transportation, demonstrating that carefully planned flaps with Ilizarov distraction is safe.

Many centres prefer staged surgery for complex infections and often separate orthopaedic and plastic surgeries (Metsemakers et al., 2019; Lowenberg et al., 1996; Pesch et al., 2020). In some cases, it may be possible to treat the bone defect and soft tissue loss by acute shortening or angulation to allow wound closure (Rozbruch et al., 2006; Pierrie and Hsu, 2017), but this was not possible in these cases. The combined use (usually staged) of Ilizarov method with free flaps has been reported, in several series. Lowenberg and Githens (2015) described the technique with good results in the management of severe open tibial fractures. However, they stressed the need for repeated debridements to eradicate infection, before the definitive Ilizarov method and free flap to reconstruct the bony and soft tissue defects.

**Figure 5:**
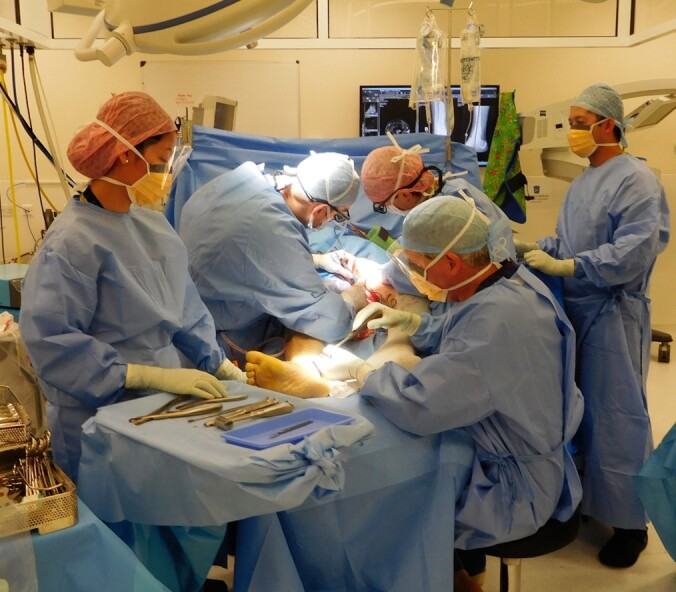
Two teams work together to harvest a gracilis muscle flap from the left thigh and excise an infected non-union from the right tibia.

This series demonstrates that Ilizarov method with free muscle flap coverage is effective in a single procedure, without prior debridement of infection. Careful planning is required between the orthopaedic and plastic surgeons and this can be facilitated by reviewing new patients together in a multidisciplinary clinic. To reduce theatre time, two scrub teams are employed so that the remote flap can be harvested whilst the osteomyelitis excision and frame application proceed (Fig. 5).

Free flap procedures for chronic osteomyelitis are often more challenging with loss of normal tissue planes due to chronic inflammation and scarring. Axial vessels can be disrupted from previous injury with increased spasticity. The literature available on free flap failure rate in the treatment of chronic osteomyelitis is sparse but has been quoted from 0 %–19 % (Chan et al., 2019; Lowenberg et al., 1996; McKee et al., 2008; Gonzalez and Weinzweig, 2005; Chim et al., 2011). The flap failure rate of 3/57 (5.3 %) is no higher than that quoted for flaps used for chronic bone infection, without frames. All flap complications occurred in the few hours after flap transfer prior to any frame adjustments. It was notable that Ilizarov distraction around or under a flap had no effect on flap survival. No flaps had complications after 72 h and this coincides with the time at which the microvascular anastomosis has endothelialised. Muscle flaps were preferred in this series due to the evidence suggesting faster bone union compared to fasciocutaneous flaps (Chan et al., 2012). Post-operatively, muscle flap compression using Coban self-adherent bandaging (3M™, Saint Paul, Minnesota, USA), was used to reduce flap swelling whilst mobilizing in the frame.

Corticotomy for lengthening or bone transport was undertaken in most cases (84.2 %) at index procedure. In our opinion, corticotomy may be delayed if: (1) the level of the planned corticotomy is very close to the vascular anastomosis or (2) there is pan-diaphyseal infection, such as after removal of an infected tibial nail. In these cases, the medullary dead space was filled with dissolving local antibiotic pellets (Herafill G; Heraeus Medical GmbH, Wehrheim, Germany). In cases with associated angular deformity the corticotomy was delayed for around 6 weeks, if made at the level of the osteomyelitis segment. Using this approach, no infections occurred in the regenerate.

Bony union was successfully achieved in all cases. However, this required an additional 10 procedures (five during frame treatment and five after frame removal) (mean 0.17 operations per patient). The frame index was 1.5 months / cm${}^{-1}$ in the bone transport group, surprisingly shorter than the 2.1 months cm${}^{-1}$ in the ASR group. This difference may be explained by the higher number of unplanned surgeries performed in the bone transport group to secure docking site union and remove the frame earlier (0.89 additional procedures per patient with bone transport, compared to 0.3 per patient with ASR). These results with bone union are similar to other series of Ilizarov method in infected tibial non-union with minimal use of flaps (Tetsworth et al., 2017; Eralp et al., 2016; Khan et al., 2015).

There are many benefits of single stage definitive surgery, principally that it offers a more patient-friendly treatment compared with other published treatment options. It allows the start of recovery immediately, as opposed to the increased treatment duration incurred by staging intervention. Patients can plan for the operation with less uncertainty around staging and timing of treatment. The consequent reduced hospital length of stay results in reduced healthcare utilisation and lower costs (Lowenberg et al., 2013; Sharma et al., 2015; Shirley et al., 2018; Olesen et al., 2016). Yet, there are significant resource implications in delivering single stage surgery, not least in the need for long operative time and combined orthoplastic teams who are familiar with this complex surgery. It may be difficult to identify sufficient theatre time to perform this surgery as a single stage, but overall, avoiding multiple stages reduces the total theatre time per patient.

This study is limited by the retrospective assessment of outcome. A mean follow-up of almost 6 years is reasonable but clearly infection can recur many years after surgery, so our results may underestimate the final recurrence rate. This paper reports a significant group of patients from a single specialist centre, which may not be comparable with other groups. However, all consecutively presenting patients were recruited to avoid selection bias. This treatment algorithm should be applied within the more general and comprehensive recommendations for managing osteomyelitis and fracture-related infections (Metsemakers et al., 2019; BMJ, 2020; Govaert et al., 2020).

## 5 Conclusion

This study shows that with the right expertise, simultaneous debridement, Ilizarov frame and free muscle flap transfer is safe and effective in treating complex limb infection. It requires organisation of services for effective delivery and careful preoperative planning. The results show that correctly performed distraction osteogenesis techniques do not adversely affect free flaps and are not associated with an increased flap failure rate, or reduced bone healing.

## Ethical statement

This study was conducted in a tertiary healthcare centre, providing specialty care to patients with musculoskeletal infections. The study was performed in accordance with the Declaration of Helsinki and approved as an audit of clinical practice by the hospital governance board (OUH 2020 6400).

Patients gave written consent for their images to be used as figures. In all cases, the identity of the patient has been protected.

## Data Availability

Study data is currently stored in a database which contains patient identifiable information. Our institutional policy on information governance and national healthcare system prevents access to data which may allow identification of individual patients.
